# Narrative Matters: *Adolescence* in The Manosphere – A perfect storm?

**DOI:** 10.1111/camh.70012

**Published:** 2025-07-14

**Authors:** Jane Gilmour

**Affiliations:** ^1^ The UCL Great Ormond Street Institute of Child Health University College London London UK; ^2^ Great Ormond Street Hospital for Children NHS Foundation Trust London UK

**Keywords:** Toxic masculinity, online safety, adolescence, adolescent brain development, radicalisation

## Abstract

The Netflix drama ‘Adolescence’ brought ‘the manosphere’ (a virtual space defined by misogynist views) and its potential dangers for teenage boys into the mainstream narrative. This paper describes parallels between toxic masculinity communities and other extremist groups. It considers why so many teenage boys may be attracted to the manosphere and why a developmental context is crucial to an informed response. The unique characteristics of ‘the teenage brain’ offer a framework to guide professionals working with young people who are exploring this type of content.

The Netflix series *Adolescence* got the world talking about ‘the manosphere’: a virtual space defined by misogynist views and toxic masculinity. Mainstream media has the power to propel issues to the top of the political and social agenda, bringing with it an opportunity for professionals and academics to leverage the spotlight and inform the debate. So, what can we add?

*Adolescence* describes a 13‐year‐old boy, Jamie, who fatally stabbed a female classmate. As the drama unfolds, we learn that he had been bullied by his peers and rejected by the girl he murdered. Crucial to our understanding of Jamie, he felt neglected by his Dad, a man with a violent childhood who set out to avoid aggression in his own family, and yet both he and Jamie shared a volatility – simmering below the surface. Jamie's parents are portrayed as loving but uninvolved and distant from his emotional life. For some months before the attack, he had been left to his own devices exploring the internet, alone in his bedroom. Themes specifically linked to toxic masculinity percolate the narrative: with the strong implication that Jamie had been radicalised by the manosphere.

The origins of the manosphere movement are more than 50 years old. Some argue that the principles of Men's Rights Activists were a reaction to Women's Lib, and others argue that misogyny has been ever thus. But the zeitgeist is notable; contemporary themes in other spheres share values with the manosphere. For example, there are parallels between some populist political policies and the principles of toxic masculinity: both create a scapegoat which – if diminished in power or number, will redress the ‘natural balance’ and deliver optimum living circumstances. Perhaps predictably, radical politics and misogyny are comfortable bedfellows in the cyber world, Mamié, Ribeiro, and West (2021) described 300 million internet posts and established that ‘auto suggestions’ pushed those engaging in the manosphere towards extreme ‘Alt‐Right’ content.

These data are undoubtedly unsettling, but as professionals invested in the wellbeing of young people, our concern is heightened when we consider the demographic consuming the material. In part because some manosphere content is not on mainstream channels and in part because of the significant time required to gather and publish high‐quality evidence, we do not yet have adequate empirical data to describe consumption patterns. Those data that do exist suggest 80% of 16‐ and 17‐year‐old boys have seen manosphere content (Hope Not Hate survey, 2023). Developmental theorists may argue that the manosphere and the adolescent brain create a perfect storm. Consider the unique brain state of a postpubertal teenager: searching for an identity, prone to take risks and explore unknowns, highly sensitive to perceived status, driven to be accepted and respected by peers and adult society (see Steinberg & Morris, 2001, for review) and primed for associative learning experiences (Towner, Chierchia, & Blakemore, 2023). These drives add up to a vulnerable brain perfectly placed to consume, learn and potentially internalise toxic masculinity.

One of the reasons that adolescents and young men enter the manosphere so frequently is that it very often includes fitness tips, wealth generation schemes (likely to be fraudulent) luxury cars and so on. These topics are – traditionally at least – attractive to adolescent boys. Baker, Ging, and Brandt Andresen (2024) described creating dummy accounts for hypothetical teenage boys searched for traditional generic masculine content, such as #gymtips, and others for manosphere content, such as #redpill (indicating ‘a realisation’ that society discriminates against men). Both types of account search found the manosphere in almost equivalent time, whether they searched using #redpill or #gymtips. As they explore the digital world, adolescent boys may not be looking for misogyny but because it is wrapped up in content that interests them, online misogyny is very likely to find them. Understanding the lure of ‘digitally adjacent’ content, such as fitness, may offer clues about how young people may avoid harmful misogyny without losing the benefits of the digital world.

Of course, not all young men exposed to this content will be affected by it or agree with it but the Hope Not Hate survey (2023) suggests that only 26% of 16‐ to 17‐year‐old boys rejected the ideas of one particular manosphere content provider and 45% considered them positively. A proportion of this latter group may be at risk of internalising the ideals or in extreme cases, becoming radicalised. Radicalisation is a gradual process, but it is often triggered by a personal event. In Jamie's case, rejection by the girl in his year ‘confirmed’ his growing belief that he was disempowered, disregarded and neglected by society and his hero Dad. The psychological literature offers limited data on the risk factors linked to misogynist extremism, but there are established patterns described in extremist religious or political groups. Adolescence itself is a risk factor but in addition, social disconnection, family dysfunction, a sense of abandonment or inequality and poverty are examples of characteristics that alone or in combination that increase the chances of extremism (see Campelo, Oppetit, Neau, Cohen, & Bronsard, 2018 for review). Manosphere proponents use recruitment techniques recognisable from extremist groups, such as capitalising on a perceived injustice (‘*feminism is a cancer’* – women's rights dominate society at the cost of men), fear mongering (‘*80% of women want to date 20% of men’* – only ‘Alpha Males’ with manosphere values) and isolation (‘*stay lonely to be successful’* – social relationships slow down achievement).

Unsurprisingly, the current debate includes an urgent call for practical strategies to protect young men and boys from these harmful influences. There is a proposal that *Adolescence* be shown in all secondary schools with the implication that simply viewing it would be an effective prevention or intervention. Without specialised facilitation, it is not clear what message we are expecting to send young people, particularly as many of the adult characters' responses are inadequate. For example, Jamie's parents were characterised as feeling helplessness. *What else could we have done? We thought he was safe in his room…*but helplessness does not invite effective action. The investigating police officer in the series dismisses his own teenage son's valuable insights and in doing so missed a chance to reconnect to him, losing the most valuable of tools in any intervention: a good relationship. The forensic psychologist was portrayed as lacking basic clinical skills and was not able to contain Jamie's feelings safely. *Adolescence* is a great drama but not a policy document. Below are suggestions for an informed framework and response for clinicians and other professionals working with young people:

## A developmental approach

This paper argues that a developmental approach, drawing on established adolescent brain literature, will offer evidence‐based principles to develop interventions. The very characteristics that make adolescents vulnerable to the pernicious nature of the manosphere are the same characteristics that can offer a data‐led approach.

## Consultation

Adolescents have an increased sensitivity to social status hierarchy so using a consultative model is a useful principle in any communication with teenagers. Professionals and parents alike will recognise that didactic instruction will rarely change an adolescent's attitude or behaviour, likely because lecturing does not convey mutual respect to a young person so highly attuned to social position. Evidence from a variety of health behaviour initiatives indicate that positive change is more likely if we offer young people contextual information and with appropriate facilitation, they use their developing autonomy to shift towards healthier choices (see Yeager, Dahl, & Dweck, 2018 for review). In the case of the manosphere, consider supporting young people to investigate, using alternative and legitimate sources, the motivation of high profile content providers (defrauding their followers) or Big Tech (commoditising users' attention).

## Curiosity

Ask young people who are interested in the manosphere what they think of the content with genuine curiosity and without shaming them. This is important for two reasons; first using a respectful tone changes the odds of engagement (see Yeager et al., 2018 for review), but crucially, it is more likely to deliver information about the *true* function of exploring content for each young person. Is it an interest in luxury cars, fitness regimes, a search for powerful language, a way out of poverty or is it, in the worst case, a validation of misogynist views? Asking open questions may reveal that the content is consumed *despite* the content providers' attitudes to women. Given the adolescent brain's sensitivity to associative learning, there is a risk that ‘incidental’ consumption of toxic views may become normalised or internalised, so it is critical that the adults supporting young people help them establish *credible* alternative sources for their preferred content, outside the manosphere.

## Identity experimentation

Young adolescents characteristically explore identities, often in extreme ways, before inhabiting a more moderate, stable self. This is a healthy developmental process in most instances, but it means adolescent boys may explore the identities inhabiting the manosphere. Some adolescents who identify with a toxic ‘Alpha Male’ persona may in fact be connecting with specific character attributes that are not inherently misogynistic, while others may be in the manosphere precisely because it is testing boundaries in the mainstream narrative. Clinical anecdotal experience indicates that many teenage boys want to feel powerful and *that* underlies their perceived connection with manosphere content providers. Trusted adults can help young men disentangle different aspects of the manosphere character and explore what attracts them. In this example, it might be important to note that power is not a zero‐sum game; it is possible to feel a sense of potency without diminishing women and girls.

## Alternative role models

There is a developmentally appropriate pull away from the biological family post puberty, so while parents will continue to be highly influential, social learning theory points to the power that high status, older role models can have whether they are online or not. Policy makers are starting to recognise the need for mentors in the community, but in addition, professionals can guide young people towards youth workers or sports coaches, for example. Regular opportunities to be alongside relatable and aspirational figures who are respectful of women will help shift the narrative for vulnerable young people.

## Risk taking

Adolescents are more likely to take risks, particularly when they are around their peers. Risk means that the outcome is unknown and emerging data indicate that the same individuals are likely to take both negative (engaging with dangerous online content) and positive risks (successfully going for an audition) (Duell & Steinberg, 2020). The context dependant nature of risk taking offers an intervention strategy because the type of risky choices can be shaped by peers, family and the wider community. Armed with this knowledge, adults can steer young people towards taking chances that are likely to have a positive impact on their trajectory.

*Adolescence* invited an important discussion about how to support teenage boys who risk consuming toxic masculinity beliefs. Effective strategies must include a developmental framework capitalising on the specific characteristics of the adolescent brain; but interventions cannot operate in a social vacuum. It is striking that Jamie had many unsatisfactory relationships with family, friends and the adults around him. Perhaps more public messaging and policy initiatives should start by highlighting the fundamental and ubiquitous value of building good relationships with young people.

If you are concerned about a young person's safety or the safety of others, further support can be found here https://www.gov.uk/guidance/get‐help‐if‐youre‐worried‐about‐someone‐being‐radicalised

## Funding information

There are no funders to report for this paper.

## Conflict of interest statement

The author declares that there are no conflicts of interest in relation to this article.

## Ethics statement

Not applicable for this Narrative Matters paper.

## Data Availability

Data sharing is not applicable to this article as no new data were created or analysed.
